# “Health Is in the Air”: Visual and Olfactory Nudges and Food Choices at University Vending Machines

**DOI:** 10.3390/nu18193292

**Published:** 2026-10-07

**Authors:** Simone Chiarelli, Chiara Casiraghi, Alessandra Cecilia Jacomuzzi, Valeria Micheletto, Vincenzo Russo, Margherita Zito, Giuseppina Gifuni

**Affiliations:** 1 Department of Business, Law, Economics and Consumer Behaviour “Carlo A. Ricciardi”, Università IULM, 20143 Milan, Italy; simone.chiarelli@studenti.iulm.it (S.C.); chiara.casiraghi@iulm.it (C.C.); valeria.micheletto@iulm.it (V.M.); vincenzo.russo@iulm.it (V.R.); margherita.zito@iulm.it (M.Z.); giuseppina.gifuni@studenti.iulm.it (G.G.); 2 Behavior and Brain Lab IULM—Neuromarketing Research Center, Università IULM, 20143 Milan, Italy; 3 Department of Philosophy and Cultural Heritage, Ca’ Foscari University of Venice, 30123 Venice, Italy

**Keywords:** nudge, olfactory cues, visual cues, food choice, consumer behavior, vending machines, healthy eating, university students, sensory marketing

## Abstract

**Background/Objectives**: Ambient sensory cues can influence food choice, yet their role in everyday environments remains underexplored, with much of the existing evidence derived from laboratory studies. This is particularly relevant in university settings, where students often make rapid, convenience-driven choices that may be nudged toward healthier options by sensory cues (e.g., images or scents). **Methods**: A quasi-experimental field study was conducted across three consecutive one-week conditions: control, visual (a nature-themed poster), and olfactory (jasmine fragrance). Snacks were classified according to perceived healthiness ratings from a pre-study questionnaire. After predefined exclusions, purchases were analyzed using binary logistic regression, with condition as the main predictor and price and time of day examined as additional factors. **Results:** Among *N* = 1043 purchases, products classified as healthy were more frequent during the olfactory week than during the control and visual weeks. The visual condition did not differ significantly from control. Higher prices were associated with lower odds of purchasing a product classified as healthy, whereas time of day was not significantly associated with purchasing behavior. **Conclusions:** These findings provide field-based evidence suggesting that olfactory cues may be associated with a shift toward food choices perceived as healthier in vending-machine environments, pointing to their potential as a low-cost sensory nudging strategy while highlighting the need for confirmation through randomized or counterbalanced designs and replication across diverse real-world food-choice settings beyond university contexts.

## 1. Introduction

Making food choices is far from simple. It is a complex decision-making process arising from the interaction of sensory, psychological, and physiological factors with environmental cues, habits, and food accessibility [1]. Modern lifestyles characterized by high stress, time constraints, and convenience-oriented eating further shape our daily food decisions [2]. These pressures are particularly evident among university students, whose choices are influenced by academic demands and the availability of food on campus [3,4,5,6]. In this setting, vending machines provide accessible snacks and beverages, although the options available may be nutritionally limited [7,8].

Promoting healthy food choices has thus become a central goal in global health agendas [9], as unhealthy diets are a major modifiable risk factor for non-communicable diseases [10,11]. However, designing effective interventions in real-world contexts can be challenging. Nutritional knowledge alone may be insufficient to ensure healthy behavior, because food-related decisions reflect the interaction of individual motives and features of the immediate choice environment [12,13].

In line with dual process models of decision making, food choices can involve affective, associative, and heuristic processes alongside reflective, information-oriented reasoning [14]. These processes may give rise to biases such as the “unhealthy = tasty” intuition [15]. Previous food experiences also contribute to expectations and preferences: sensory associations stored in memory can be activated when a product is encountered, while food images can elicit affective responses even without conscious awareness [16,17]. Food-related experiences thus involve cognitive and emotional responses across different sensory phases [18], and modifications of the choice environment through sensory cues can shape consumer evaluations and behavior [19].

According to the Stimulus–Organism–Response (S-O-R) model [20], external sensory cues may elicit affective and cognitive reactions, which then guide behavioral responses. From a Schema Theory perspective [21], such cues may also activate pre-existing food-related representations linking sensory features (e.g., scents, colors, or textures) with product characteristics. When an activated schema fits the product category, processing may be facilitated in line with Processing Fluency Theory [22]. Sensory nudges can therefore be understood as modifications of the choice environment that may influence food perception and choice without restricting the available options and preserving individual freedom [23].

Among the sensory features of food environments, visual and olfactory cues emerge as particularly relevant. Unlike tactile cues, which generally require physical contact, both can be introduced around a vending machine without altering the products or the purchasing procedure [19]. Visual cues can direct attention and influence food-related expectations and choices [24,25,26]. In particular, a nature-themed poster placed beside a vending machine was associated with increased purchases of snacks perceived as healthy [27]. Exposure to images of natural environments has also been linked to healthier food choices in experimental settings [28]. Olfactory cues may influence food-related behavior through associations and affective processes [29,30,31]. Prolonged exposure to indulgent food scents has been associated with lower selection of unhealthy items [32], while a laboratory study found that a jasmine scent reduced chocolate craving relative to the control odor [33]. These studies provide a rationale for examining the two modalities, although craving ratings and choices made under controlled conditions do not necessarily predict actual vending-machine purchases.

Despite these findings, evidence on sensory nudges in everyday vending machine settings remains limited. Existing interventions have primarily focused on product placement, labeling, product availability, consumer awareness, and visual cues [26,27,34,35,36,37,38], while multisensory approaches have only recently been examined under controlled conditions [39]. Their association with actual purchases in everyday university settings therefore remains underexplored.

While previous research has provided insights into how sensory cues influence food choices, few studies have examined their impact within real-world contexts, such as university settings. Most field experiments have been conducted in school cafeterias, primarily targeting product placement or labeling (e.g., fruit positioning, shelf prominence, or calorie displays [34,35]), while vending machines, one of the most frequent sources of (unhealthy) snack consumption in university contexts, have received less attention.

One of the few studies on vending machines has demonstrated that a nature-related visual nudge placed next to a real machine increases healthy snack purchases, compared to a hedonic poster [27]. Recently, a black vending machine wrap was found to result in significantly more caffeine-based selections [38], while health-oriented visual cues have been shown to steer consumers toward low-calorie beverages [26]. While this evidence highlights the potential of visual environmental cues in shifting consumer behaviors, their real-world impact remains modest and context-dependent, as their effectiveness often diminishes under conditions of time pressure [40], reduced visual attention, or habitual decision-making [41]. Similarly, pleasant scents have been demonstrated to elevate mood and decrease stress [42], mitigating emotional eating patterns common among university students, but olfactory nudging evidence in vending settings is still very limited. Lately, both visual and olfactory nudges have been found to enhance cognitive and affective neural responses and promote healthier snack selection in a simulated vending-machine context [39]. However, the study was conducted under controlled laboratory conditions, limiting the ecological validity of its effects in real-world contexts.

Finally, further variables like price and time were considered. Higher prices are generally associated with lower food purchases, and healthy foods are no exception [43], while consumers may also associate healthier foods with higher prices [44]. Pricing interventions, including discounts on healthy products, can thus promote healthier purchasing [45]. Time of day may also influence food choice, with unhealthy products being purchased more frequently in the evening [46].

Given the potential of visual and olfactory cues to influence food perception and choice, the present study examines whether a nature-themed poster and a jasmine scent are associated with shifts toward snacks perceived as healthier in a real-world university vending-machine setting. It extends field-based evidence on sensory nudging while examining two interventions that leave the available products and their prices unchanged.

### Research Objectives & Hypotheses

Previous studies suggest that visual and olfactory cues may be associated with healthier food choices, yet their associations with actual purchases in vending machines remain unclear. Building on the reviewed evidence, this study examines visual and olfactory cues separately in a university vending-machine setting, compared to a no-cue control condition. Three hypotheses were developed.

**H1.**  *Exposure to nature-themed sensory cues (either visual or olfactory) will be associated with a higher likelihood of selecting snacks perceived as healthy compared to a control condition. *

A nature-related image is expected to be associated with a higher likelihood of choosing healthy-classified snacks (H1a), consistent with the evidence that exposure to such stimuli enhances the selection of low-calorie items [27,28]. Likewise, jasmine scent is expected to be associated with a higher likelihood of choosing healthy-classified snacks (H1b), as research has previously shown that non-food, “relaxing” scents can reduce cravings for high-calorie foods [33].

**H2.**  *The olfactory condition will be associated with a higher likelihood of selecting snacks perceived as healthy compared to the visual condition.*

Time pressure can alter the effectiveness of food-choice nudges [40]. Visual cues may also depend on their salience [41], while olfactory cues could act through more implicit and affective mechanisms that bypass deliberate cognition. Moreover, from a neurophysiological point of view, olfactory processing directly engages limbic regions involved in emotion, reward, and memory [29,30], suggesting stronger implicit effects than visual cues.

**H3.**  *Economic and temporal variables will also be associated with snack choices.*

Purchased products with higher prices are expected to be associated with a lower likelihood of being perceived as healthy rather than unhealthy (H3a), consistent with evidence that price can influence food purchasing decisions, including the purchase of healthier products [43,45]. Later times of day are expected to be associated with more indulgent choices, as fatigue and stress may reduce self-regulation [3] (H3b), although such temporal effects appear modest in vending contexts [46].

## 2. Materials and Methods

The study analyzed real-world purchase data recorded by a vending machine located in a university campus building. To ensure ecological validity, the sample was not actively recruited or pre-selected but comprised all individuals who spontaneously completed a purchase during the study period. Before the intervention phase (described in Section 2.2), the products sold by the vending machine were classified into two categories (healthy vs. unhealthy) based on perceived healthiness (described in Section 2.1). The study was conducted in accordance with the Declaration of Helsinki and was approved by IULM University IRB (see the Institutional Review Board Statement for further details). Since the study relied exclusively on fully anonymized purchase data generated by the vending machine and no personal data, identifiable information, video recordings, or direct interaction with participants were involved, the IRB waived the requirement for informed consent.

### 2.1. Preliminary Product Classification

Following the approach of similar studies [39], product classification into “healthy” and “unhealthy” was based on perceived healthiness, selected as the most ecologically valid operationalization of product healthiness in this context [27,37]. Perceived healthiness was assessed using the Health dimension of the Food Choice Questionnaire [47]. It included six items, which were first translated into Italian and then back-translated. In the questionnaire, participants first viewed the product images and rated each item on a Likert scale (1 = strongly disagree, 6 = strongly agree), omitting a neutral midpoint to discourage neutral responses [48]. Both product images and questionnaire items were presented in random order to control order effects. The questionnaire was distributed online, yielding 201 responses (82% F, 18% M; age = 19–69 years, *M* = 24.73, *SD* = 11.33). Responses from individuals with food intolerances, allergies, or vegan diets were excluded to avoid bias in perceived healthiness ratings for products containing animal-derived ingredients (i.e., most sweet products), resulting in a final sample of 149 participants (80% F, 20% M; age = 19–69 years, *M* = 26.66, *SD* = 12.95). Internal reliability of the Health dimension was excellent (α > 0.90). To ensure balanced categorization and in line with prior research examining perceived food healthiness [39,49], products were then split and classified as healthy or unhealthy according to the sample median perceived healthiness rating. The evaluated items corresponded to the standard commercial assortment of packaged snacks and cold beverages regularly stocked in the vending machine prior to the study. To ensure that observed purchasing patterns were not driven by variations in product availability across conditions, only the 25 products consistently available across all three experimental weeks were retained for the analyses (Table 1). A *t*-test indicated a statistically significant difference between products classified as healthy and those classified as unhealthy, with higher mean ratings for the healthy group (*t*(24) = 6.11, *p* < 0.001).

### 2.2. Sensory Interventions

The study took place over three consecutive weeks in November 2024 (November 4–23) and included three intervention conditions, implemented in the following way: Control (baseline condition, with no sensory intervention; November 4–9), Visual (November 11–16), and Olfactory (November 18–23). The period was selected to avoid exam sessions, holidays, or extended breaks that could systematically alter students’ purchasing behavior. To further support the representativeness of this period, overall sales recorded during the study were compared with sales data from the same period in the previous two years, indicating that purchasing volumes during the study period were comparable. The sequential order was determined by the operational constraints of the naturalistic setting. Specifically, the olfactory condition was placed last to minimize potential carryover from residual fragrance into following conditions. While this choice reduced the risk of sensory contamination, some of the observed differences may also reflect variation across weeks, leading the study to be considered a quasi-experimental field design rather than a fully randomized experiment.

Purchases from an actual vending machine located at Magazzino 5 building, San Basilio campus of Ca’ Foscari University of Venice, Italy, were analyzed. The machine was the one placed in the highest-traffic area frequently visited by undergraduate and master’s students. Students attending the building were expected to be 19–26 years old, mainly female, and enrolled in humanities programs. These are contextual estimates: building access and individual exposure could not be recorded, so the specific demographics of people who encountered the intervention could not be determined.

The break area offered a relatively neutral and low-stimulus environment, featuring only the vending machine, water dispensers, and plain walls, minimizing the presence of competing visual or contextual cues that could interfere with or dilute the impact of the nudge interventions (e.g., other posters). The machine was filled with products such as sweet snacks (e.g., cookies, chocolate bars, fruit bars, candies), savory snacks (e.g., crackers, breadsticks, chips), and beverages (e.g., juices, soft drinks, ready-to-drink iced coffee). Over the three intervention weeks, the vending machine setup was kept as stable as possible, including product assortment, product positioning, pricing, and restocking procedures. From the first to the third week, the following interventions were implemented, each lasting one week:

- Control. No sensory intervention was introduced, and the vending machine and surrounding area were maintained under their usual operating conditions.
- Visual. Consistent with prior research suggesting that visual cues may be associated with healthier food choices [27], a nature-themed poster (50 × 70 cm), supported by an easel, was positioned at eye level on the right side of the vending machine and kept in the same position throughout the intervention week. In terms of size, color, contrast, and brightness, the images were matched to the specifications reported in previous studies [27].
- Olfactory. In line with previous research suggesting that exposure to jasmine scent may reduce cravings, particularly for unhealthy foods [33], jasmine fragrance oil (Mystic Moments, Fordingbridge, Hampshire, UK) was diffused near the vending machine using an aroma diffuser (Pure Aroma 150 Yang, Cecotec, Valencia, Spain) placed out of sight behind the vending machine. The diffuser was activated every day approximately 30 min before university opening [39] and operated continuously throughout opening hours, using the same diffusion settings (continuous mode) on all days of the olfactory condition. The vending area was an indoor space with mechanical ventilation, which remained unchanged throughout the intervention week. To ensure the interventions were consistent and maintained, researchers regularly monitored the vending machine several times a day to verify that the stimuli of the interventions were correctly positioned and, during the olfactory condition, that the diffuser was operating with the intended settings. Moreover, a close collaboration with the vending company ensured regular monitoring of the machines throughout the data collection period, so that no technical issues could occur, either with the machine itself or with the purchase records.

### 2.3. Data Analysis

The study examined whether the proportion of purchases classified as healthy versus unhealthy differed across three experimental conditions (Control, Visual, and Olfactory), each implemented during a separate one-week period. Purchase transactions were extracted from the vending machine logs. In collaboration with the vending company, machine functionality and log completeness were monitored throughout the study period, with no malfunctions or missing-record issues reported.

To ensure comparability across conditions, the dataset (*N* = 4343 purchases across the three weeks) was cleaned to include only packaged snack and beverage items that had been classified in the preliminary product classification procedure. Purchases of products introduced after the start of the study or not available in all three weeks were excluded (*N* = 473). Water and beverages prepared on demand (tea, coffee, cappuccino, hot chocolate) were also excluded (*N* = 2827), as they do not involve packaging cues relevant to perceived healthiness and their composition may vary (e.g., added sugar). Water was excluded because it was also freely available from a dispenser whose use was not recorded, so bottled water purchases would have captured only part of participants’ choices. These exclusions yielded a final analytic sample of *N* = 1043 purchases.

To test our hypotheses, binary logistic regression models were estimated in JASP (version 0.95.0) [50], with Product Category (0 = Unhealthy; 1 = Healthy) as dependent variable. Condition was entered as a categorical predictor with Control as the reference category (M_1_). Despite unequal numbers of observations across conditions, logistic regression was appropriate given the sufficient number of outcome events relative to the predictors included [51]. In M_2_, Price was entered as a continuous item-level covariate associated with each purchase. A third model (M_3_) additionally included Time of day, categorized into four time windows based on the purchase timestamp (7–10 a.m. = morning; 10–12 a.m. = late morning; 12–4 p.m. = afternoon; after 4 p.m. = evening). Model comparisons were performed using likelihood-ratio tests and information criteria (AIC/BIC). Because adding Time of day did not improve model fit (∆χ^2^ = 2.88, *p* = 0.411) and resulted in less favorable information criteria, the main analyses focused on M_2_. To directly test H2, a planned contrast comparing the Olfactory and Visual conditions was performed within M_2_.

## 3. Results

In the Control condition, 395 purchases were recorded: 179 (45.3%) classified as healthy and 216 (54.7%) classified as unhealthy. In the Visual condition, 396 purchases were recorded: 177 (44.7%) classified as healthy and 219 (55.3%) classified as unhealthy. Finally, in the Olfactory condition, 252 purchases were recorded: 214 (84.9%) classified as healthy and 38 (15.1%) classified as unhealthy (Table 2).

Logistic regression models were estimated with Product Category (0 = unhealthy; 1 = healthy) as the dependent variable (Table 3).

Model fit indices showed that M_2_ provided the best fit to the data (AIC = 1255.98, BIC = 1275.78), with a significant improvement over M_1_ (∆χ^2^ = 54.38, *p* < 0.001).

Regarding M_2_ coefficients (Table 4), the Olfactory Condition (*β* = 1.941, *SE* = 0.211, *z* = 9.188, *p* < 0.001, OR = 6.96, 95% CI [4.61, 10.53]) was significantly associated with a higher likelihood of selecting a product classified as healthy. In contrast, among completed purchases, higher prices of the selected products were associated with a lower likelihood that the purchased product was classified as healthy rather than unhealthy (*β* = −1.599, *SE* = 0.240, *z* = −6.666, *p* < 0.001, OR = 0.20, 95% CI [0.13, 0.32]). The Visual Condition did not have a significant effect (*p* = 0.584).

A direct contrast between the Olfactory and Visual conditions showed that the Olfactory condition was associated with significantly higher odds that the purchased product was classified as healthy rather than unhealthy (*β* = 2.021, *SE* = 0.212, *z* = 9.544, *p* < 0.001, OR = 7.55, 95% CI [4.98, 11.43]).

## 4. Discussion

In the context of vending machines, where students frequently select unhealthy options, unobtrusive interventions offer a promising avenue to foster healthier habits. Our study investigated whether nature-themed sensory cues (a poster and a jasmine fragrance) were associated with shifts in university vending-machine purchases toward snacks perceived as healthier. Across a three-week intervention (control, visual-nudge, and olfactory-nudge weeks, and 1043 valid purchases analyzed), we tested whether nature-themed cues would increase healthy choices relative to control (H1), whether the olfactory cue would exert a stronger shift than the visual cue (H2), and how economic and temporal factors related to snack choice (H3).

Our findings provide partial support for H1. The two nature-themed conditions related differently to choice patterns: the jasmine scent (olfactory cue) was associated with a significantly higher proportion of purchases classified as healthy compared to the control condition, supporting H1b and aligning with evidence that ambient scent can alter food-related behavior [32,52]. In contrast, the nature-themed poster (visual cue) was not significantly associated with snack selection relative to control, failing to support H1a.

The pattern observed between modalities supported H2: the olfactory condition yielded significantly higher odds of healthy purchases than the visual condition. This finding aligns with evidence that olfactory stimuli can evoke immediate, unconscious responses that influence food preferences [39]. Neurally, olfactory processing directly engages limbic pathways involved in emotion and memory below conscious awareness [30]. By contrast, visual nudges depend more heavily on attentional engagement. The nature poster may thus not have been sufficiently salient to alter habitual choices, although visual attention and cue awareness were not measured. These findings suggest that visual and olfactory cues may influence food choices differently even when they convey similar semantic associations. Nonetheless, the magnitude of the olfactory association warrants caution given the quasi-experimental design. The olfactory week recorded fewer total purchases than the control and visual weeks. Because the analyses included only completed transactions, they cannot determine whether the scent influenced product choice itself, the likelihood of approaching the machine, or in the composition of purchasers. Thus, the higher proportion of healthy purchases may partly reflect selection or week-specific factors rather than product choice alone.Results also clarified the role of situational constraints (H3), indicating a stronger association for economic than temporal factors. Supporting H3a, among completed purchases, higher prices of selected products were associated with a lower likelihood of being classified as healthy rather than unhealthy. This is consistent with rational choice accounts and previous evidence showing that food purchasing is price-sensitive, particularly for healthier products [43,45,53]. However, because prices were not experimentally manipulated and the model included only completed transactions, this association should not be interpreted as an estimate of price elasticity or as evidence that higher prices reduced overall purchasing propensity. Rather, price represents an important item-level characteristic associated with the perceived healthiness of the purchased product. Finally, H3b was not supported, as snack choices did not vary significantly across time windows. This stability may reflect students’ irregular schedules, where diurnal fatigue patterns diverge from standard work routines, or indicate that vending machine purchases are driven primarily by immediate hunger or hedonic impulses regardless of the hour [46].

### 4.1. Potential Attentional Differences Between Visual and Olfactory Cues

A central question is why the visual intervention failed to shift snack choices, despite positive findings in similar contexts [27,39]. Because the break area offered a neutral, low-stimulus environment, visual clutter is an unlikely explanation. Instead, this null result likely reflects boundary conditions inherent to visual nudging in habitual environments.

In fast or routine settings such as vending machines, consumers frequently display inattentional blindness, overlooking peripheral visual cues as their attention is focused on the desired product or keypad [41]. Visual nudges may also compete with salient product displays and situational distractions such as smartphone use or peer interactions [54,55]. While unmeasured here, these represent plausible contextual barriers to the effectiveness of the visual intervention.Individual and situational differences also affect cue responsiveness [56]. Visual nudges are typically more effective when health goals are already active: field evidence shows that visual cues support healthy choices primarily among consumers with pre-existing health goals [57]. In a general university population with heterogeneous health goals, visual cues may fail to engage everyone. Additionally, cue specificity is critical: while aligned, congruent visual elements yield stronger behavioral shifts, while generic or incongruent stimuli often produce null results [58,59]. Here, the nature poster may have conveyed broad semantic associations of freshness rather than direct, actionable product-level signals.

The stronger association during the olfactory condition may finally suggest that olfactory and visual cues operate through distinct mechanisms in this setting. Olfactory processing involves direct connections with limbic structures supporting emotion and associative memory with minimal cognitive filtering [30]. Furthermore, ambient scents operate in the background and can influence behavior without deliberate processing [60]. In line with evidence guiding our scent selection [32,33,52], the jasmine fragrance may have activated health-congruent associations and attenuated cravings without requiring the overt visual attention that consumers frequently bypass during rapid, habitual choices.

### 4.2. Theoretical and Practical Implications

Theoretically, these findings can be interpreted within multisensory decision-making models, extending sensory nudging research through ecologically situated evidence from real vending machine transactions. Consistent with the S-O-R framework [20], environmental sensory cues may precede behavioral responses, though intervening affective and cognitive states remain plausible mechanisms rather than directly measured pathways. Furthermore, from Schema Theory [21] and Processing Fluency Theory [22] perspectives, the jasmine scent may have functioned as a perceptual cue congruent with pre-existing health schemas, subtly increasing the perceptual fluency and salience of healthy items without restricting consumer freedom. Direct schema activation, however, was not assessed. Finally, these findings contribute to Nudge Theory [61] highlighting the role of atmospheric and sensory features of the choice environment, and showing that sensory modalities perform differently under habitual, time-constrained conditions.

Practically, sensory features offer viable public-health tools in food-choice environments where decisions are rapid and deliberation is minimal. Ambient scents provide an unobtrusive intervention that operates without requiring overt visual attention, a distinct advantage when consumer attention is fragmented or focused strictly on products. Conversely, the null visual finding suggests that visual cues may be less effective in routine retail settings when consumers do not directly attend to them. While replication is needed before broad implementation, these insights offer preliminary guidance for designing sensory interventions in university vending environments.

### 4.3. Limitations and Future Research Directions

While offering field-based evidence on sensory nudging, several limitations contextualize these findings and guide future research.

First, total purchases dropped during the olfactory condition compared to the control and visual conditions. Because analyses included only completed transactions, they capture choices conditional on a purchase occurring, but cannot determine whether the scent influenced foot traffic, the propensity to approach the machine, or the demographic composition of consumers. Although checks with the vending operator confirmed no technical malfunctions or stock-outs occurred, and unequal sample sizes do not invalidate logistic regression, the large effect size warrants caution. Unmeasured contextual factors (e.g., class schedules) may have amplified this shift. This association should thus not be viewed as a pure modality-specific effect and requires replication with balanced transaction volumes.

Second, conditions were implemented sequentially rather than randomized or counterbalanced to avoid fragrance carryover. Although data collection took place during a stable academic period to minimize temporal bias, unobserved week-to-week variation (e.g., fluctuations in attendance, academic deadlines, or weather) cannot be excluded. This limits causal inference, as part of the olfactory effect may stem from temporal variation. Future research should employ randomized crossover sequences, repeated intervention cycles, or multi-machine installations to isolate causal effects.

Third, data were collected from a single machine in an Italian university, limiting generalizability to other populations or contexts where jasmine may hold different meanings, and public venues (e.g., workplaces, transit stations, hospitals). Future research should replicate these findings across multiple sites and explore possible effects on nearby food outlets, such as campus cafeterias.

Moreover, each condition lasted only one week, precluding conclusions regarding long-term persistence. While repeated exposure might induce sensory adaptation and diminish nudging efficacy, it could also foster lasting habits. Longitudinal studies spanning several months with alternating intervention and baseline periods are needed to evaluate temporal stability. Furthermore, product categorization relied on survey-based perceived healthiness ratings, consistent with previous food-choice research [27,39,49] and reflecting consumer perceptions within this setting [27,37]. However, subjective perceptions may diverge from objective nutritional profiles (e.g., a “healthy” fruit bar might be high in sugar). Moreover, while median-based classification aligns with prior studies in this context [39,49], dichotomizing perceived healthiness simplifies product variation and makes borderline items sensitive to the chosen cutoff. Thus, our findings reflect shifts toward products perceived as healthier rather than objective nutritional improvements. This interpretation is also limited to the products analyzed: bottled water was excluded, and unrecorded usage of the freely available water dispenser prevented capturing water selections or substitution effects across conditions. Future research should integrate standardized nutritional indicators (e.g., Nutri-Score, energy density, or sugar content) alongside subjective perceptions [62] and, where feasible, record water selections for a more complete choice overview.

Regarding the scents, only jasmine was examined. As olfactory priming depends on odor-specific semantic associations and hedonic valence [52,60], these findings cannot be generalized to olfactory nudges broadly. Future research should compare diverse fragrances (e.g., citrus, lavender) alongside neutral or control scents to determine whether specific olfactory profiles evoke distinct food-choice patterns.

In addition, anonymous transactions precluded tracking repeated purchases, potentially introducing non-independence among observations. Additionally, institutional privacy regulations prevented collecting purchaser demographics, leaving the exact proportion of students versus staff unverified across conditions. Future studies should implement individual tracking (e.g., student cards or mobile apps) to account for within-subject clustering via mixed-effects modeling.

Finally, because the study evaluated the real-world effectiveness of ambient nudges rather than their mechanistic efficacy, direct manipulation checks and other ambient-related exposure monitoring were omitted to preserve ecological validity. Consequently, while capturing unprompted purchasing behavior, the data cannot establish whether the shift stemmed from conscious perception, implicit processing, or individual variations in olfactory sensitivity. Future research could combine field studies with controlled laboratory experiments to explore the mechanisms underlying these effects, using manipulation checks, exposure measures, and neurophysiological or attentional metrics [39,63].

## 5. Conclusions

This field study suggests that ambient sensory cues may be associated with distinct food choices in university vending-machine settings. The jasmine-scent condition was associated with a higher proportion of purchases classified as healthy than both the control and visual conditions, whereas the nature-themed visual cue was not associated with a significant shift. Higher prices were associated with a lower likelihood that purchased products were classified as healthy. Time of day was not significantly related to choice. The findings extend sensory nudging research to an ecologically valid food-choice setting and highlight the potential relevance of olfactory cues in shaping everyday consumer decisions and supporting healthier food choices. Future research should build on these findings and test the robustness of these effects across different settings, populations, and sensory cues, and further examine the mechanisms through which sensory nudges may influence food-related decisions.

## Figures and Tables

**Table 1:** Perceived healthiness scores (*M*) of the *N* = 25 snacks presented in the vending machine and analyzed. Snacks categorized as “healthy” are highlighted in green.

Product Name	Product Type	Perceived Healthiness (M)	Product Category
Product 1	Croissant	9.10	Healthy
Product 2	Chips	7.81	Unhealthy
Product 3	Breadsticks	8.52	Healthy
Product 4	Crackers	13.11	Healthy
Product 5	Chocolate candies	7.24	Unhealthy
Product 6	Rice crackers	11.79	Healthy
Product 7	Crackers	13.66	Healthy
Product 8	Crackers	10.81	Healthy
Product 9	Breadsticks	10.29	Healthy
Product 10	Chocolate bar	7.44	Unhealthy
Product 11	Yogurt muffin	9.07	Healthy
Product 12	Fruit bar	14.52	Healthy
Product 13	Breadsticks	11.15	Healthy
Product 14	Chocolate wafer	8.18	Unhealthy
Product 15	Chocolate bar	7.78	Unhealthy
Product 16	Soft drink	6.55	Unhealthy
Product 17	Candies	7.64	Unhealthy
Product 18	Coffee beverage	7.64	Unhealthy
Product 19	Coffee beverage	7.45	Unhealthy
Product 20	Energy drink	6.57	Unhealthy
Product 21	Chocolate bar	7.30	Unhealthy
Product 22	Rice cookie	8.72	Healthy
Product 23	Juice	8.36	Healthy
Product 24	Soft drink	8.02	Unhealthy
Product 25	Fruit bar	13.48	Healthy
*Median*		*8.36*	

**Table 2:** Distribution of purchased Product Category (0 = unhealthy; 1 = healthy) across experimental conditions.

Condition	Product Category	Frequency	Percentage
Control	0	216	54.7%
	1	179	45.3%
	Total	395	100%
Visual	0	219	55.3%
	1	177	44.7%
	Total	396	100%
Olfactory	0	38	15.1%
	1	214	84.9%
	Total	252	100%

**Table 3:** Model summary with Product Category as dependent variable. M_1_ includes Condition; M_2_ includes Condition and Price; M_3_ includes Condition, Price, and Timing.

Model	Deviance	AIC	BIC	*df*	∆χ^2^	*p*	Nagelkerke R^2^
M_0_	1437	1438.871	1443.821	1042	134.508	–	0.000
M_1_	1302	1308.363	1323.213	1040	134.508	<0.001	0.162
M_2_	1248	1255.983	1275.782	1039	54.380	<0.001	0.222
M_3_	1299	1311.483	1341.182	1037	2.880	0.411	0.165

**Table 4:** Logistic regression coefficients for M_1_ and M_2_. Product Category levels: 0 = unhealthy; 1 = Healthy. SE = Standard Error; OR = Odds Ratio.

Model	Predictor	Estimate	SE	OR	z	Wald	*p*
M_0_	Intercept	0.187	0.062	1.205	2.999	8.995	0.003
M_1_	Intercept	−0.188	0.101	0.829	−1.859	3.456	0.063
	Condition (Olfactory)	1.916	0.203	6.796	9.441	89.134	<0.001
	Condition (Visual)	−0.025	0.143	0.975	−0.175	0.031	0.861
M_2_	Intercept	1.292	0.239	3.641	5.404	29.200	<0.001
	Condition (Olfactory)	1.941	0.211	6.964	9.188	84.427	<0.001
	Condition (Visual)	−0.080	0.147	0.923	−0.547	0.299	0.584
	Price	−1.599	0.240	0.202	−6.666	44.442	<0.001

## Data Availability

The datasets generated and analyzed during the current study are not publicly available but are available from the corresponding author upon reasonable request.
